# Supplementary material for: Chloroplast Localized FIBRILLIN11 Is Involved in the Osmotic Stress Response during Arabidopsis Seed Germination
Source: Biology (Basel). 2021 Apr 25;10(5):368. doi: 10.3390/biology10050368 (PMC8145590; doi:10.3390/biology10050368)
Supplement: Supplementary file 1 [file biology-10-00368-s001.zip › biology-1169968-supplementary.pdf]

**Supplementary Data**

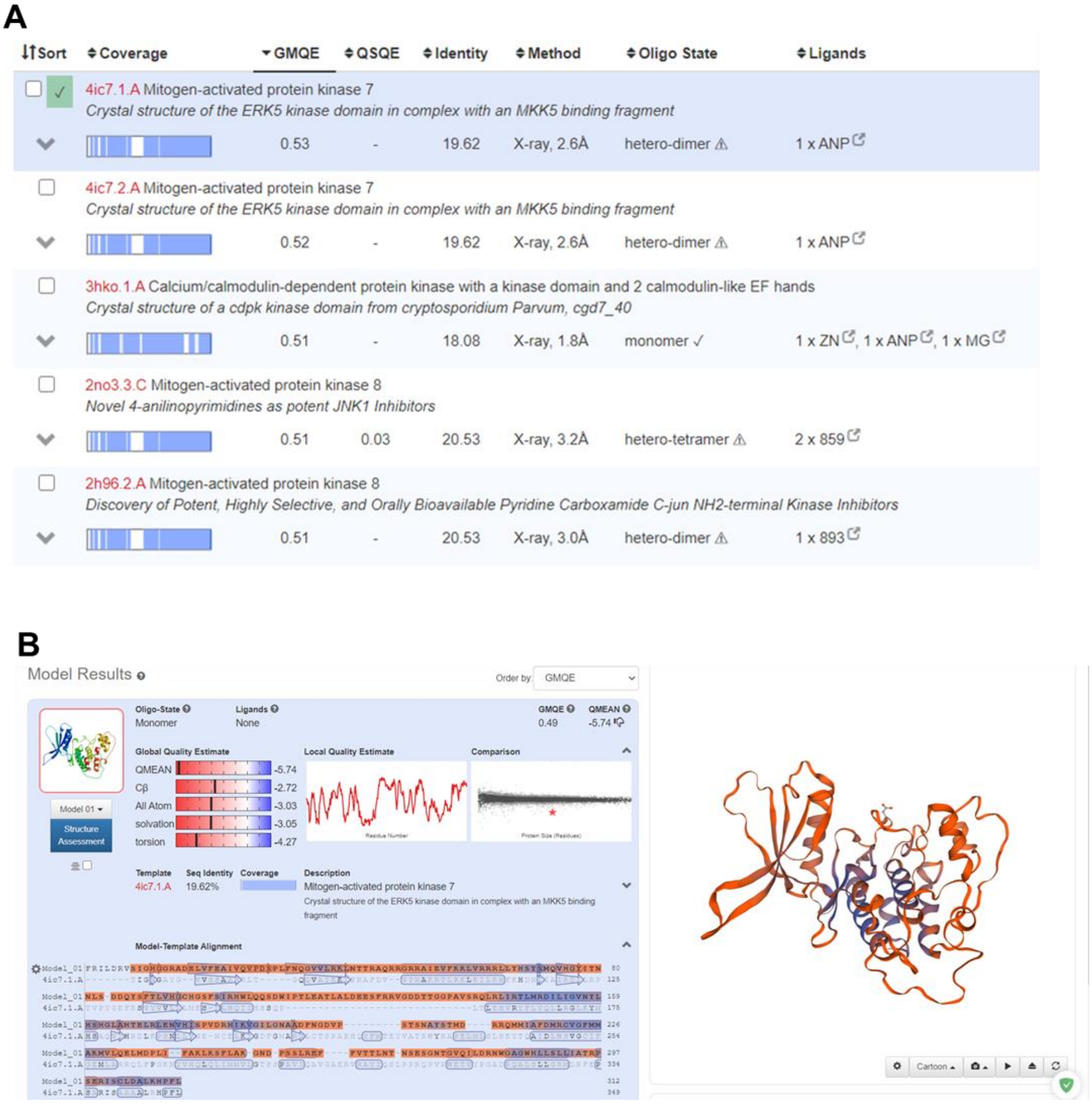


**Figure S1.** Tertiary structure analyses of the FBN11 kinase domain using SWISS-MODEL (A) List of Top 5 proteins with structures most similar to the FBN11 kinase domain (B) Tertiary structure model of the kinase domain of FBN11 based on the tertiary structure of mitogen-activated protein kinase 7

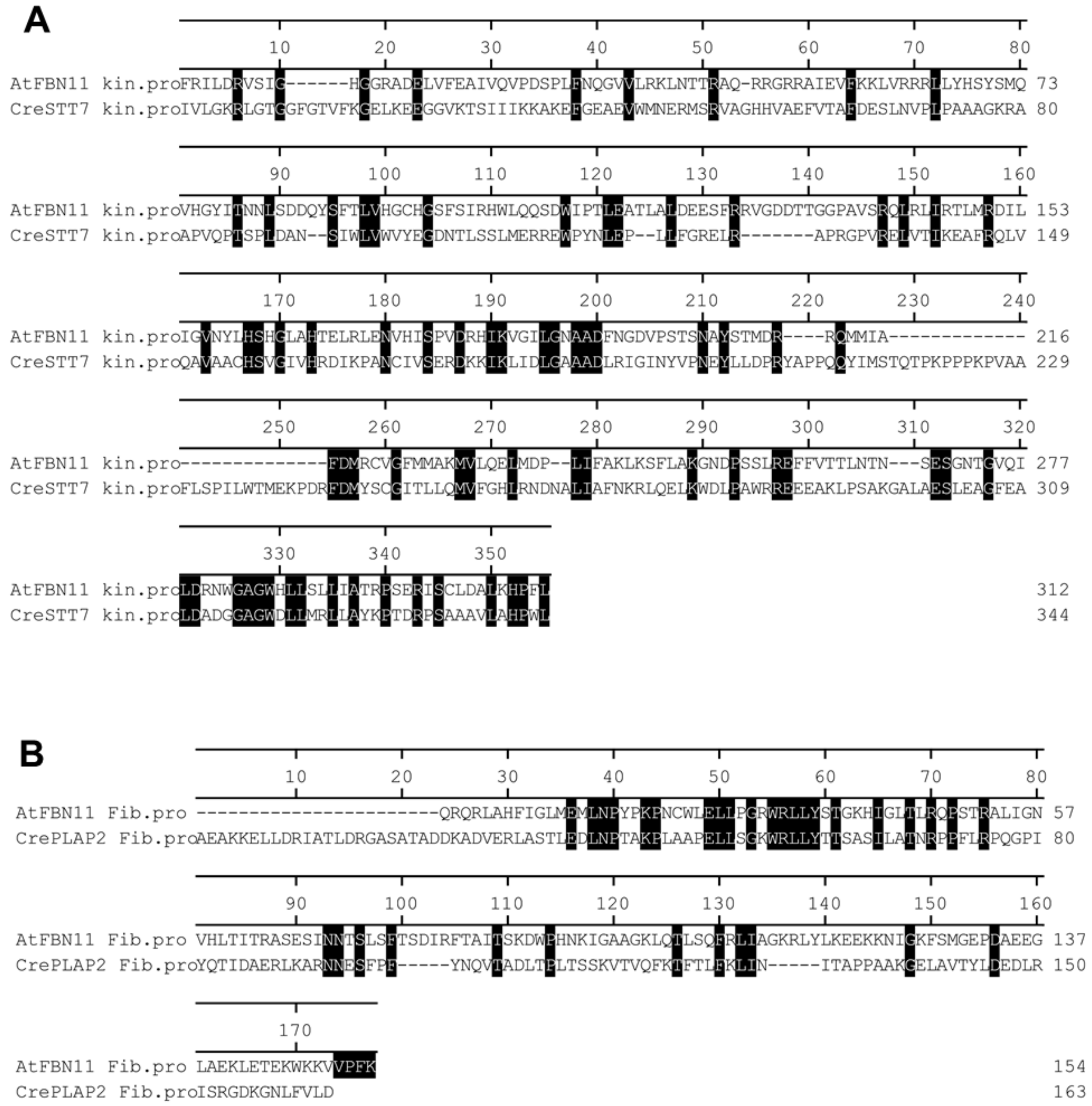


**Figure S2**. (A) Amino acid alignment between the kinase domain of Arabidopsis FBN11 and CreSTT7 of Chlamydomonas. (B) Amino acid alignment between FBN11 and the CrePLAP2 FBN domain of Chlamydomonas.

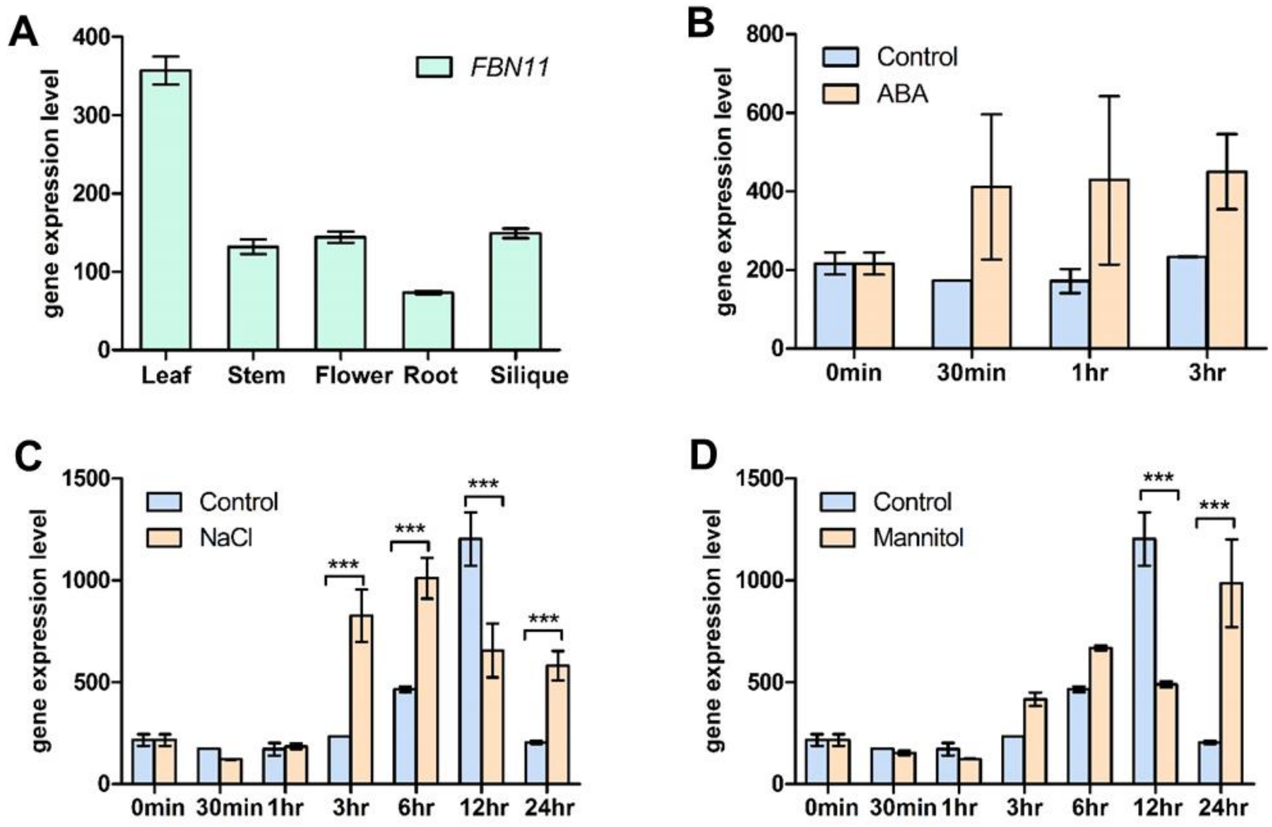


**Figure S3.** *FBN11* gene expression profiles in various tissues under various abiotic stresses. (A) *FBN11* gene expression levels in leaves, stems, flowers, roots, and silique of wild-type Arabidopsis. (B) Effect of 10 μM ABA treatment on *FBN11* gene expression levels in 7-day-old Arabidopsis seedlings. (D) Effects of 150 mM NaCl (C) and 300 mM mannitol (D) on *FBN11* gene expression levels in 18-day-old Arabidopsis seedlings. Statistical analysis is done by two-way ANOVA with Bonferroni post-test (***$P<0.001$).

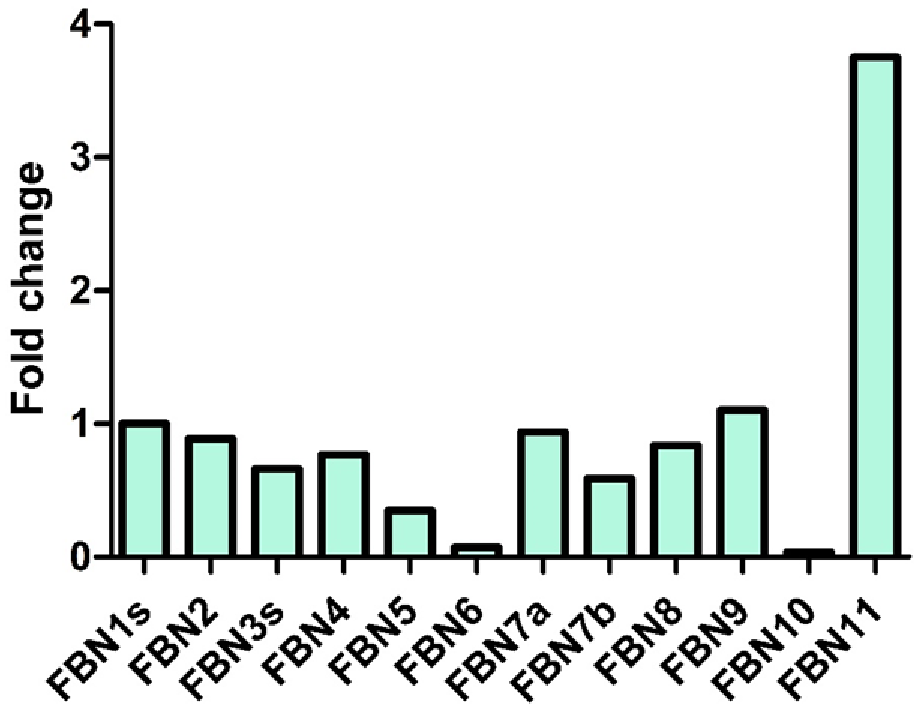


**Figure S4**. Expression profiles of *FBN* family genes in stage 10 seeds without siliques. This analysis was performed with data from the TAIR eFP browser (http://bar.utoronto.ca/efp2/Arabidopsis/Arabidopsis_eFPBrowser2.html). The expression value of each gene is a fold change based on *FBN1a*. *FBN1s* represents *FBN1a* and *1b*. *FBN3s* stands for *FBN3a* and *3b*. These duplicated genes are indistinguishable because of their high sequence homology.

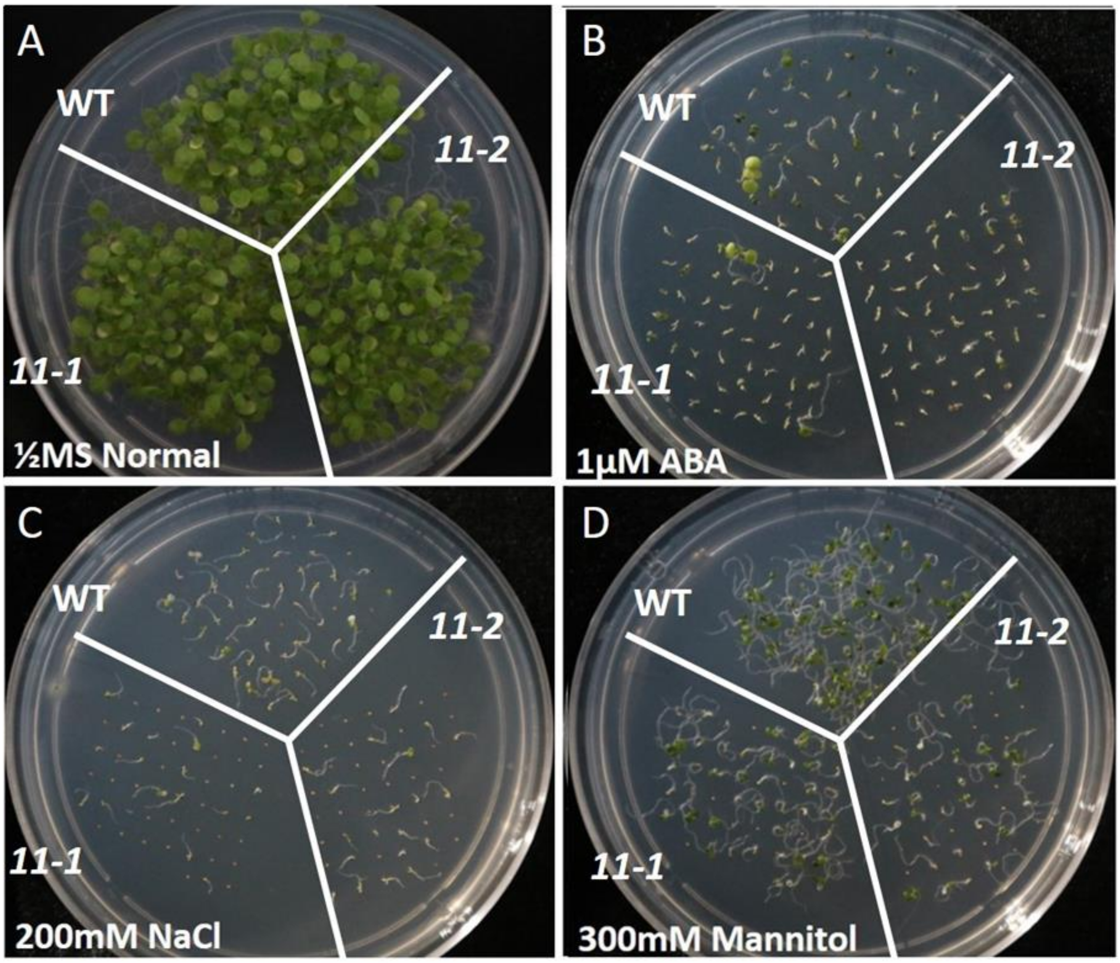


**Figure S5**. Seed germination phenotypes of wild type and *fbn11* homozygous mutants under stress. Unsupplemented growth media (A), or media supplemented with 1 µM ABA (B), 200 mM NaCl (B), or 300 mM mannitol (D). Representative photographs of 7- day old plants.

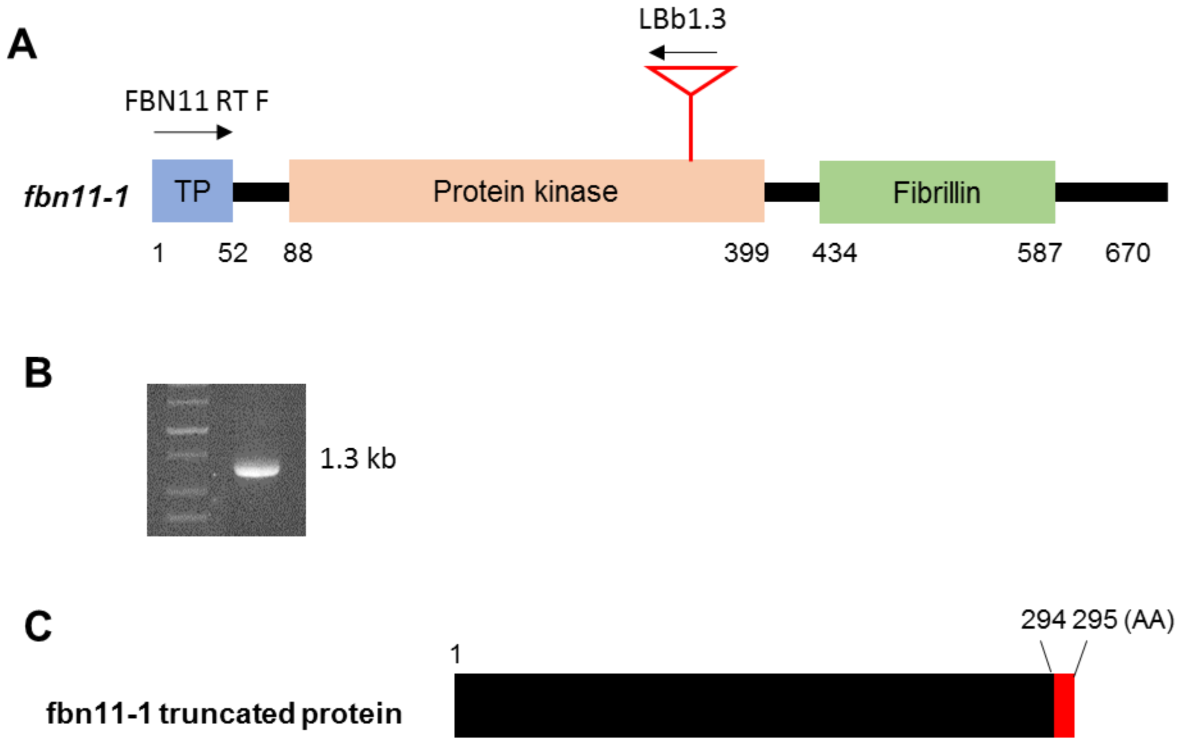


MQFKWSDFRILDRVSIGHGGRADELVFEAIVQVPDSPLFNQGVVLRKLNTTRAQRRGRRAIE
VFKKLVRRRLLYHSYSMQVHGYITNNLSDDQYSFTLVHGCHGSFSIRHWLQQSDWIPTLEATLA
LDEESFRRVGDDTTGGPAVSRQLRLIRTLMRDILIGVNYLHSHGLAHTELRLENVHISPVDRHIK
VGILGNAADFNGDVPSTSNAYSTMDRRQMMIAFDMRCVGFMMAKMVLQELMDPLIFAKL
KSFLAKGNDPSSLREFFVTTLNTNSESGNTGVQILDRNWGAGWHQ

**Figure S6.** *fbn11-1* truncated transcript and deduced protein sequences. (A) The T-DNA insertion site of *fbn11-1* and primers position for truncated transcript detection (B) Transcript detected by RT-PCR from RNA of *fbn11-1* seedlings using FBN11 RT F and LBb1.3 primer (Table S1) (C) Protein sequence (295AA) encoded by *fbn11-1* truncated transcript. The black box represents the wild-type FBN11 amino acid sequences, and the red box represents the amino acid sequence generated by T-DNA fusion.

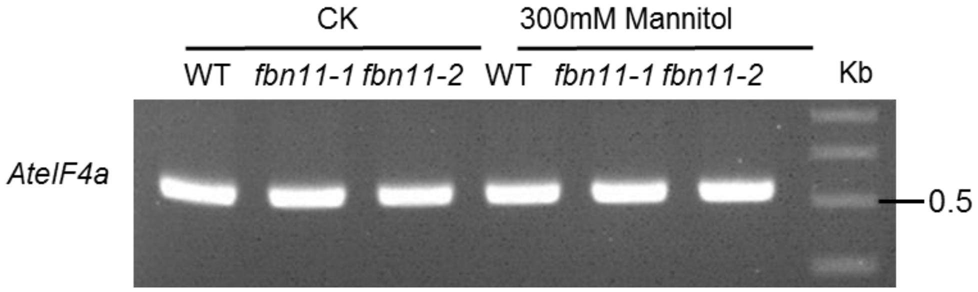


**Figure S7**. RT-PCR expression analysis of *AteIF4* during seed germination of wild type and *fbn11* mutants non-treated (CK) and treated with 300 mM mannitol. PCR with 30 cycle amplification was performed using primers (forward: 5'-CGTGGTTTCAAGGACCAGAT-3' and reverse: 5'-ATTGATCGCAACACCCTTTCT-3') that detect 0.5 kb of *AteIF4* transcript.

**Table S1.** Primers used in this study.

| Primer name | | Sequence |
|---|---|---|
| | Relative expression for ABA pathway gene | |
| qRT-ABF2 | F | 5'-ACATACCAGCAATCGCAACA-3' |
| | R | 5'-CCACAAGACCACCACCTCTT-3' |
| qRT-ABF4 | F | 5'-TGGTCAGCCAAATCAAAACA-3' |
| | R | 5'-GTTGTTGTTGCTGCTGCATT-3' |
| qRT-ABF1 | F | 5'-GCCTGGAGAAGGTTGTTGAG-3' |
| | R | 5'-TCAGCTTCCAGTTCCAAGGT-3' |
| qRT-ABF3 | F | 5'-CTGGAAGGAGCTGATGAAGG-3' |
| | R | 5'-AGCCCTGACCAAAAACTCCT-3' |
| qRT-DREB2A | F | 5'-GGGTAAATGGGTTGCTGAGA-3' |
| | R | 5'-CGAGCCAAAGGACCATACAT-3' |
| qRT-CBF3 | F | 5'-ACAGAGGAGTTCGTCGGAGA-3' |
| | R | 5'-CCATCTCAGCGGTTTGAAAT-3' |
| qRT-CBF1 | F | 5'-CTACGAATCCCGGAGTCAAC-3' |
| | R | 5'-ATCGTCTCCTCCATGTCCAG-3' |
| qRT-CBF2 | F | 5'-AACAGAGCCAAGATGCGTTT-3' |
| | R | 5'-ACGTCATCATCTCCCTCGAC-3' |
| | Identification for T-DNA insertion mutants | |
| *fbn11-1* | LP | 5'-TATTAAGGCACGTGTGGAAGG-3' |
| | RP | 5'-GCAACGCTTACAGTACCATGG-3' |
| *fbn11-2* | LP | 5'-CGAATTTTCAAACCCTAAATCG-3' |
| | RP | 5'-TTGTAAATTCGGCAGATTTGG-3' |
| LBb1.3 | genotyping | 5'-ATTTTGCCGATTTCGGAAC-3' |
| FBN11 RT | F | 5'-ATGGCACTTTGTGGTGTTT-3' |
| | R | 5'-CTACATAGACTTATGGTCCAAG-3' |
| | Generation of complementary plant | |
| pDONR-FBN11 | F | 5'-AAAAAGCAGGCTGTATGGCACTTTGTGGTGTTTG-3' |
| | R | 5'-AGAAAGCTGGGTGCATAGACTTATGGTCCAAGCTG-3' |
| qRT-FBN11 | F | 5'-AGCCAAACTGTTGGTTGGAG-3' |
| | R | 5'-ATTAAGGCACGTGTGGAAGG-3' |
| GFP | R | 5'-TGAACTTGTGGCCGTTTACG-3' |
| | R(full) | 5'-TTACTTGTACAGCTCGTCC-3' |

**Table S2.** Amino acid composition and secondary structure of FBN11.

| Amino acid composition | |
|---|---|
| Ala (A) | 5.1% |
| Arg (R) | 7.8% |
| Asn (N) | 4.5% |
| Asp (D) | 4.8% |
| Cys (C) | 1.5% |
| Gln (Q) | 3.1% |
| Glu (E) | 5.1% |
| Gly (G) | 6.4% |
| His (H) | 3.3% |
| Ile (I) | 6.0% |
| Leu (L) | 11.3% |
| Lys (K) | 4.8% |
| Met (M) | 3.1% |
| Phe (F) | 4.2% |
| Pro (P) | 4.5% |
| Ser (S) | 9.9% |
| Thr (T) | 4.9% |
| Trp (W) | 1.8% |
| Tyr (Y) | 1.9% |
| Val (V) | 6.1% |
| **SOPMA secondary structure prediction** | |
| Alpha helix (Hh) | 32.09% |
| Extended strand (Ee) | 19.25% |
| Random coil (Cc) | 44.33% |

**Table S3.** FBN11 physical parameters.

| | |
|---|---|
| Number of amino acids | 670 aa |
| Molecular weight | 75,766. Da |
| Theoretical pI | 9.43 |
| Instability index | 49.17 |
| Aliphatic index | 90.34 |
| Grand average of hydropathicity (GRAVY) | -0.242 |
| Number of predicted TMHs | 0 |
| Number of AAs in TMHs | 0.03 |

**Table S4.** FBN11 orthologs analysis of plant evolution

| Green plants | species | common name | FBN11 ortholog (Locus) | AA identity (%) |
|---|---|---|---|---|
| Dicots* | *Arabidopsis thaliana* | Thale Cress | AT5G53450 | 100 |
| | *Brassica rapa* | Mustard | Bra003055 | 88 |
| | *Carica papaya* | Papaya | evm.model.supercontig_3.70 | 69 |
| | *Cucumis sativus* | Cucumber | Cucsa.040680.2 | 65 |
| | *Glycine max* | Soybean | Glyma04g41510.1 | 70 |
| | | | Glyma06g13320.1 | 68 |
| | *Lotus japonicus* | Lotus | Lj1g0007393.1 | 70 |
| | *Manihot esculenta* | Cassava | cassava4.1_003020m | 68 |
| | *Mimulus guttatus* | Mimulus | mgv11b005497m | 64 |
| | *Medicago truncatula* | Barrel medic | Medtr3g091290.1 | 68 |
| | *Ricinus communis* | Ricinus | 30169.m006496 | 69 |
| | *Prunus persica* | Peach | ppa002468m | 67 |
| | *Populus trichocarpa* | Poplar | POPTR_0015s01910.1 | 69 |
| | | | POPTR_0492s00210.1 | 63 |
| | *Solanum tuberosum* | Potato | PGSC0003DMP400016058 | 64 |
| | | | PGSC0003DMP400016057 | 62 |
| | *Vitis vinifera* | Wine Grape | GSVIVG01038710001 | 67 |
| Monocots* | *Brachypodium distachyon* | Brachypodium | Bradi2g45450.1 | 59 |
| | *Oryza sativa* | Rice | LOC_Os01g46720.1 | 58 |
| | *Sorghum bicolor* | Sorghum | Sb03g029870.1 | 59 |
| | *Setaria italica* | Foxtail millet | Si000637m | 60 |
| | *Zea mays* | Maize | GRMZM2G150952_T01 | 60 |
| Lycophytes* | *Selaginella moellendorffii* | Spike Moss | g105971 | 48 |
| Bryophytes** | *Physcomitrella patens* | earthmoss | Pp1s110_47V6.1 | 24 |
| | | | Pp1s159_111V6.1 | 23 |
| | *Marchantia polymorpha* | umbrella liverwort | Mapoly0022s0163.1.p | 47 |
| Charophytes** | *Chara braunii* | charophyte green algae | CBR_g39728 | 36 |
| Chlorophyta** | *Chlamydomonas reinhardtii* | Chlamydomonas | Cre02.g120250.t1.1 (STT7) | 26 |

* The protein sequence of Arabidopsis FBN11 was analyzed by BLAST in the Plant Genome Database, and the ortholog was found based on E-value $1x10^{-4.}$

**The genome database of lower plants was analyzed using Ensembl Plants.

**Table S5.** *FBN* gene family comparison between Arabidopsis and *Chlamydomonas reinhardtii*.

| *Arabidopsis thaliana* | *Chlamydomonas reinhardtii* |
|---|---|
| AtFBN1a<br>AtFBN1b<br>AtFBN2 | CrePLAP7, 8, 10 |
| AtFBN3a<br>AtFBN3b | CrePLAP2 |
| AtFBN4 | CrePLAP9 |
| AtFBN5 | CrePLAP6 |
| AtFBN6 | CrePLAP4 |
| AtFBN7a<br>AtFBN7b | x |
| AtFBN8 | CrePLAP1 |
| AtFBN9 | CrePLAP3 |
| AtFBN10 | CrePLAP5 |
| AtFBN11 | STT7 |
